# iPSCs: A Preclinical Drug Research Tool for Neurological Disorders

**DOI:** 10.3390/ijms22094596

**Published:** 2021-04-27

**Authors:** Gabriele Bonaventura, Rosario Iemmolo, Giuseppe Antonino Attaguile, Valentina La Cognata, Brigida Sabrina Pistone, Giuseppe Raudino, Velia D’Agata, Giuseppina Cantarella, Maria Luisa Barcellona, Sebastiano Cavallaro

**Affiliations:** 1 Institute for Biomedical Research and Innovation (IRIB), Italian National Research Council, 95126 Catania, Italy; gabriele.bonaventura@gmail.com (G.B.); iemmolo.rosario@gmail.com (R.I.); valentina.lacognata@cnr.it (V.L.C.); sabrina.pst@libero.it (B.S.P.); 2 Department of Biomedical and Biotechnological Sciences (BIOMETEC), Section of Pharmacology, University of Catania, 95123 Catania, Italy; peppettg@hotmail.it (G.A.A.); gcantare@unict.it (G.C.); 3 Ortho-Neuro Center HUMANITAS Istituto Clinico Catanese, 95045 Catania, Italy; giuraudino@hotmail.it; 4 Department of Biomedical and Biotechnological Sciences, Section of Anatomy, Histology and Movement Sciences, University of Catania, 95123 Catania, Italy; vdagata@unict.it; 5 Department of Pharmaceutical Science, Biochemistry Section, University of Catania, 95123 Catania, Italy; marsirin@gmail.com

**Keywords:** iPSCs, drug development, AD, ALS, PD, FRAX

## Abstract

The development and commercialization of new drugs is an articulated, lengthy, and very expensive process that proceeds through several steps, starting from target identification, screening new leading compounds for testing in preclinical studies, and subsequently in clinical trials to reach the final approval for therapeutic use. Preclinical studies are usually performed using both cell cultures and animal models, although they do not completely resume the complexity of human diseases, in particular neurodegenerative conditions. To this regard, stem cells represent a powerful tool in all steps of drug discovery. The recent advancement in induced Pluripotent Stem Cells (iPSCs) technology has opened the possibility to obtain patient-specific disease models for drug screening and development. Here, we report the use of iPSCs as a disease model for drug development in the contest of neurological disorders, including Alzheimer’s (AD) and Parkinson’s disease (PD), Amyotrophic lateral Sclerosis (ALS), and Fragile X syndrome (FRAX).

## 1. Introduction

The development of new drugs is an articulated and expensive process that proceeds through several steps, starting from target identification to molecular leading compounds discovery to reach the final approving for clinical use. Currently, a very low number of drugs reach the final steps of these stages that require about 10–12 years of research and a cost of roughly $800 million. This lack of success rate is partly due to safety problems and the inappropriate therapeutic index of new experimental compounds. Until today, preclinical drug screening has been performed on animal models to obtain preliminary information that is mainly related to farmacodinamics aspects [1]. However, modeling the full complexity of human disease phenotypes in animals may not always be completely exhaustive because of the critical species-specificity differences. Neurological disorders, in particular, still remain a big challenge in the context of disease modeling, considering the main difficulty in reproducing the multifactorial and multigenic features of human disorders, and often producing disappointing and controversial results between animal models studies and human clinical trials [2,3]. Starting from these limitations, in the last decades, the research community has focused its interest in developing more appropriate disease models able to resume the human genomic background.

The discovery and development of new technologies based on induced pluripotent stem cells (iPSCs) is currently playing an essential role for drug preclinical studies in particular for human neurological conditions, allowing to test in vitro patient-specific treatment protocols, paving the way towards personalized medicine [4].

**Figure 1:**
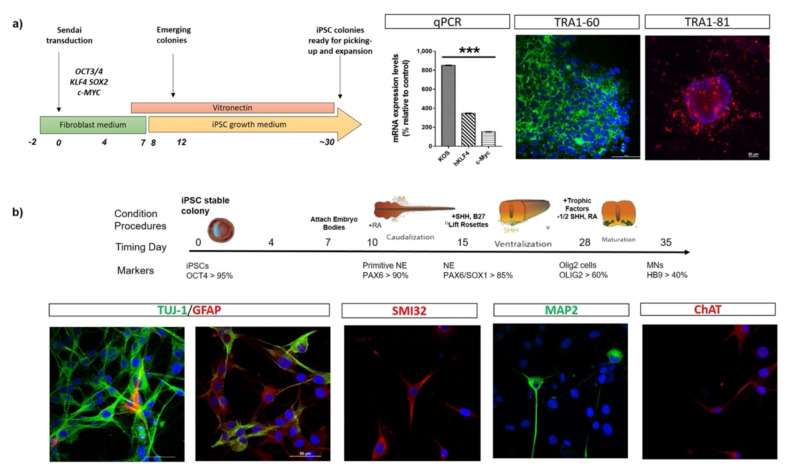
Schematic representation of cells reprogramming and neuronal differentiation in vitro. (**a**) A schematic overview of human iPSC line generation from fibroblasts. Fibroblasts are transduced with Sendai Vector (one of the non-integrating method) carrying *OCT4*, *KLF4*, *SOX2*, and *MYC*. On day 12, conditionally reprogrammed cells start to emerge in colonies expressing high levels of *KOS, KLF4*, and c-*MYC* mRNAs. On day 30, reprogrammed embryo bodies express the pluripotent stem cell markers TRA1-60 and TRA1-81, as revealed by immunofluorescence assays, and are ready for picking-up and expansion. (**b**) Representative scheme highlighting the timing of human iPSCs differentiation towards motor neuronal lineage. During neuralisation, cell morphology changes from expanding colonies to neural rosettes (NE). Cells acquire reduced cell soma area and thin extended projections connecting adjacent cells, finally differentiating into motor neurons-like cells. At the end of differentiation protocol, iPSC-derived neurons express specific neuronal markers (TUJ-1, SMI32, MAP2, ChAT).

The iPSC technology was described for the first time in 2006 by Yamanaka et al., who were able to reprogram adult somatic cells in pluripotent stem like cells by inducing the expression of four different transcription factors-encoding genes (*OCT4*, *SOX2*, *KLF4*, and *MYC)* that are responsible for the maintenance of the pluripotent stage in human embryonic stem cell (ESCs) state [5,6]. After the delivery of these transcription factors, cells lose their somatic characteristics showing morphology, gene-expression profiles, and differentiative capability analogous to human ESCs and, from that moment, can be easily pushed to differentiate into specific cell phenotypes, such as motor neurons (Figure 1).

The experimental procedures for obtaining iPSCs have been widely studied to improve the reprogramming technique, especially to ameliorate efficiency and safety. In this context, the delivering methods, initially proposed in Yamanaka et al., to introduce and integrate the transcription factors into the genome of somatic adult cells were based on retroviral vectors. This infection, performed with retroviral and lentiviral vectors, showed different disadvantages related to both the complete control on the host cell’s DNA expression and the possible tumorigenic risk that is associated with using the proto-oncogenes *KLF4* and *MYC* [7].

To overcome this critical step and avoiding or minimizing genome alterations, new methods have been introduced so far, such as non-integrating vectors (Sendai virus, Adenovirus, Episomal vectors), self-excising vectors (PiggyBac transposon), or the use of alternative molecules (Valproic acid, Vitamin C, microRNA, piRNA, and siRNA) [8].

Today, iPSCs represent a unique and well-characterized resource to elucidate neurological disease mechanisms and provide a novel human stem cell platform for screening new candidate therapeutics. Modeling human diseases using iPSCs has created novel opportunities for both mechanistic studies as well as the discovery of new disease therapies, also for neurological conditions, such as AD, PD, ALS, and FRAX. In the following sections, we provide a literature review on the use of iPSC as disease model for drug development and screening for these disorders.

## 2. iPSC-Based Drug Testing for Alzheimer Disease

Alzheimer’s disease (AD) is a progressive neurodegenerative disorder, characterized by the deterioration of cognitive functions leading to progressive inability to manage daily life. These alterations have been associated with a neuronal and synaptic depletion in hippocampus, basal forebrain, and cortex. At present, AD represents the most frequent cause of dementia (50–60% of cases), and this neurological condition is currently affecting about 24 million people worldwide with a percentage rate destined to grow in the absence of new therapeutic strategies [9]. From a neuropathological point of view, AD can be associated with the abnormal deposition of both extracellular amyloid plaques, consisting primarily of amyloid-β peptides, and intraneuronal neurofibrillary tangles, characterized by intraneuronal aggregations of hyperphosphorylated tau, a protein that regulates microtubule stabilization. This pathological context is responsible of brain atrophy and synaptic dysfunctions, leading to neuronal cell apoptosis. In particular, brain atrophy is usually recorded in the entorhinal and hippocampal cortex, which are functional areas that control cognitive and memory formation [10].

The multiple systematic linkage studies performed in the early 90′ allowed the identification of highly penetrant mutations in three main causative genes responsible for the early-onset dominantly inherited forms of AD (i.e., *APP*, *PSEN1*, and *PSEN2*, coding respectively for amyloid precursor protein, presenilin-1 and presenilin-2) [11,12,13]. This discovery radically changed our understanding of AD, opening a breakthrough about the amyloid cascade hypothesis. However, mutations in these genes explain just a small percentage of all familial AD (FAD) cases, suggesting the existence of other inherited disease-predisposing genes. Recently, Genome-Wide Association Studies allowed for the detection of novel candidate gene and inherited risk factors that were associated to FAD, such as the ATP-binding cassette, subfamily a, member 7 (*ABCA7*) [14], Clusterin (*CLU*) [15], Complement component 3b/4b receptor 1 (*CR1*) [16], Ephrin receptor (*EPHA1*) [17], Neurogenic Locus Notch Homolog Protein 3 (*NOTCH3*) [18], Sortilin-related receptor (*SORL1*) [19], and Triggering Receptor Expressed on Myeloid Cells 2 (*TREM2*) [20]. Despite these discoveries, the vast majority of AD cases (about the 75% of cases) are multifactorial late-onset sporadic forms, with no obvious familial aggregation and more than 40 loci of interest have been associated with the risk of developing AD [21]. To date, an association between sporadic, late-onset AD, and different alleles of Apolipoprotein E (*APOE*) was reported. In particular, ε4 allele was associated to an increased risk up to three-fold in the heterozygous carriers and up to 15-fold in the homozygous [22], while ε2 allele was reported as a protective factor, reducing the AD risk [23].

**Figure 2:**
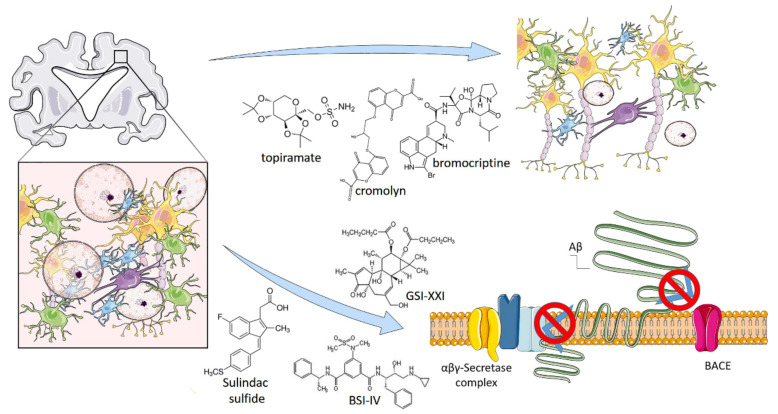
iPSC-based drug testing to treat Alzheimer’s Disease. Alzheimer’s disease (AD) is a progressive neurodegenerative disorder, characterized by an abnormal deposition of extracellular amyloid plaques. Amyloid plaques are made of insoluble β-Amyloid (Aβ) peptide deposition produced by sequential cleavages of amyloid precursor protein (APP) by b-site APP cleaving enzyme 1 (BACE1), β-and γ-secretase. Drug testing efforts conducted on AD-derived iPSC allowed to identify an anti-Aβ cocktail, composed by a mix of topiramate, cromolyn and bromocriptine, able to reduce Aβ deposition and plaques formation. Other studies have demonstrated the β-secretase inhibitor IV (BSI), γ-secretase inhibitor XXI/Compound E and sulindac sulfide efficacy in partially or fully blocked Aβ production. Illustrations used elements from Servier Medical Art (www.servier.fr/servier-medical-art).

The recent technological improvements in iPSC have allowed for the analysis of phenotypes of patients’ neural cells in vitro given the opportunity to investigate sporadic AD and test new pharmaceutical compounds, such as secretase modulators (Figure 2) [24]. In 2017, Takayuki et al. reported a selected combination of bromocriptine, cromolyn, topiramate, and anti-Aβ cocktail showing a significant anti-Aβ aggregation effects tested on human derived neurons obtained by iPSC technologies. In the first-step of the experimental screening, they analyzed a 1.258 compound library and isolated, considering inclusion and exclusion criteria, 129 compounds. In the second step, they set more stringent criteria and selected 27 compounds. Among these, six compounds (bromocriptine, cilostazol, cromolyn, fluvastatin, probucol, and topiramate) showed the maximum effect in the reduction of Aβ42. Finally, they combined these six lead-compounds by analyzing every possible combination and identified a mix of topiramate, cromolyn, and bromocriptine as the most potent combination against Aβ aggregates formation (Figure 2).

Another study was performed by Naoki e al., who differentiated from iPSC a line of neuronal cells expressing the classical neuronal markers (CTIP2, CUX1, TBR1, FOXG1, and SATB2) and the amyloid precursor protein, β-secretase, and γ-secretase components as well. They used β- and γ-secretase inhibitors (the β-secretase inhibitor IV (BSI) and γ-secretase inhibitor XXI/Compound E (GSI), respectively) and sulindac sulfide, a non-steroidal anti-inflammatory drug (NSAID). After 38 or 52 days, BSI, GSI, and NSAID partially or fully blocked Aβ production (Figure 2).

In addition, Chang et al. [25] were able to reproduce the cellular characteristics of AD using iPSCs from AD patients carrying the D678H mutation in the *APP* gene. This model enabled pharmacological screenings with an inhibitor of Aβ aggregation, the NC009-1 compound, which was able to reduce tau misfolding and improve the neuronal viability and neurite outgrowth.

In recent years, the generation of 3D cultures that were derived from iPSCs has gained a lot of interest among the research community, as it provides a human platform with higher analogies to the in vivo situation. Indeed, 3D-derived brain organoids, also constituted by neurons, astrocytes, and microglia, resemble mature human-derived tissue and represent a model with interesting applications for drug screening and discovery [26]. Some authors reported that brain organoids that use iPSCs derived from AD patients recapitulate AD-like characteristics, such as amyloid aggregation, hyperphosphorylated tau protein, and endosome abnormalities, and they are highly amenable to experimental manipulation. These patient-derived organoids have been already employed for the assessment of candidate drugs, such as (i) β- and γ-secretase inhibitors, which elicit a significant reduction in amyloid and tau pathology [27]; and, (ii) the histone deacetylase 6 (*HDAC6*) inhibitor CKD-504, which increases the degradation pathway of pathological Tau [28]. Results showed the potential of these model system for pre-clinical drug discovery in AD.

## 3. iPSC-Based Drug Testing for Amyotrophic Lateral Sclerosis

Amyotrophic lateral sclerosis (ALS) is a fatal neurodegenerative disease that is characterized by the selective and progressive loss of upper motor neurons (MNs) in the brain, and lower MNs in the brain stem and spinal cord. ALS is characterized by muscle rigidity, muscle twitching, and gradual weakness due to decreased muscle size, leading to difficulties with speech, swallowing, and, ultimately, breathing. Based on its incidence, ALS is considered to be a rare disease with approximately two cases per 100,000 individuals each year with European ancestry. The disease is male predominant, with a mean age of onset at 50–65 years and only 5% of cases manifesting an early-onset form [29]. Death occurs typically 3–5 years after diagnosis, although some forms of the disease demonstrate protracted survival [30].

Two forms of the disease have been described: familial ALS (FALS) and sporadic ALS (SALS). Familial forms represent a minority of cases (5–10%) with an autosomal dominant inheritance. Among the over 50 genes that have been linked to ALS, pathogenic mutations in Superoxide Dismutase-1 (*SOD1*), Trans-Active Response DNA-Binding Protein 43 (*TDP-43*), Fused in Sarcoma RNA Binding Protein (*FUS*), Alsin (*ALS2*), Senataxin (*SETX*), Spastic Paraplegia 11 Protein (*SPG11*), Vesicle-Associated membrane protein-associated Protein B (*VAPB*), Angiogenin (*ANG*), and Chromosome 9 Open Reading Frame 72 (*C9ORF72*) genes are the most frequently found [31,32,33,34,35]. On the contrary, the sporadic ALS form (~90% of cases) is considered to be a complex multifactorial disorder, involving defects in protein aggregation, mitochondrial dysfunction, oxidative stress, and excitotoxicity [36].

ALS is an orphan disease, as no drug or treatments are currently able to cure the disease. To date, only two compounds have been approved for ALS treatments, riluzole and edaravone, although they exert limited effects [37]. Several other pharmacologic agents have failed in clinical trials, despite promising effects in animal models. Therefore, drug discovery research for ALS has made major investments and efforts in iPSC-derived MNs modeling, as described here below.

**Figure 3:**
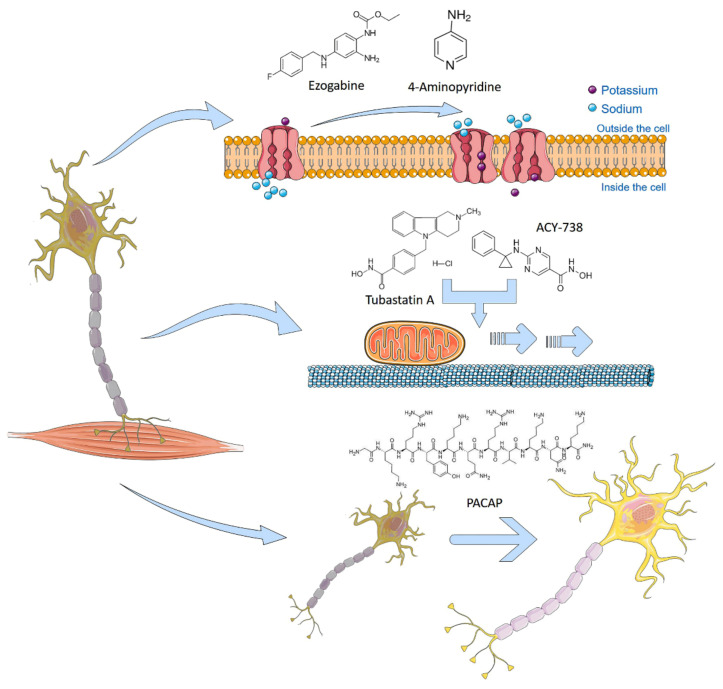
iPSC-based drug testing to treat Amyotrophic Lateral Sclerosis. ALS is considered a complex multifactorial and genetic disorder characterized by protein aggregation, mitochondrial dysfunction, oxidative stress and excitotoxicity, leading to motor neuron apoptosis. Drug testing has been conducted in order to rescue iPSC-derived affected motor neurons. The potassium channel blocker 4-Aminopyridine (4AP) or the antiepileptic ezogabine demonstrated their ability to reduce neuronal damage by restoring hypoexcitable phenotypes in *FUS*, *SOD1* or *C9ORF72* mutant iPSC-derived motor neurons. Tubastatin A and ACY-738, two specific histone deacetylase 6 (HDAC6) inhibitors, restore mitochondrial transport defects along *FUS* mutated neuronal axons. Natural peptides, such as PACAP or VIP, were shown to rescue iPSC-derived motor neurons from apoptosis, restoring mitochondrial activity and neurite outgrowth. Illustrations used elements from Servier Medical Art (www.servier.fr/servier-medical-art).

Naujock et al. observed hypo excitability in mutant *FUS* and *SOD1* iPSC-derived MNs, and investigated the effect of the FDA-approved potassium channel antagonist 4-Aminopyridine (4AP) in this model. The pharmacological treatment with 4AP decreased the potassium currents, restored spontaneous activity patterns and synaptic input, thus preventing ER stress in MNs from mutant *FUS* and *SOD1* iPSC (Figure 3). Guo et al. [38] generated iPSC-derived MNs from ALS patients with different *FUS* mutations. They reprogrammed fibroblasts from four different patients carrying two different heterozygous *FUS* mutations (R521H, P525L). These neurons showed typical cytoplasmic FUS-aggregations, altered FUS localization, as well as hypo excitability and axonal transport alterations. In order to confirm the hypo excitability hypothesis, they analyzed the electro physiological activity of ALS MNs with respect to controls, revealing a significant decrease in the frequency, but not in the amplitude, of action potentials. In addition, the patch clamp experiments showed Na^+^ peaks that were significantly lower than the controls. In the same work, they showed a FUS dependent decrease in the axonal transport of mitochondria in ALS MNs. To restore axonal transport defects, they successfully investigated the effect of α-tubulin acetylation by specific histone deacetylase 6 (HDAC6) inhibitors, such as Tubastatin A and ACY-738 (Figure 3).

Interestingly, three candidate anti-ALS drugs-ropinirole (ROPI), retigabine, and bosutinib-have been identified in iPSC-based drug screenings, and are now being evaluated in clinical trials for safety and effectiveness [39]. The first one, ropinirole hydrochloride, showed an antiapoptotic effect in iPSC-derived spinal MNs from both sporadic and familial ALS patients’ tissues. This drug, a dopamine receptor agonist, is able in improving mitochondrial activity by inhibiting TDP-43 and FUS aggregation, and reducing oxidative stress [40]. Currently, there is an on-going phase I/IIa randomized, double-blind, placebo-controlled, single-center, open-label continuation clinical trial (UMIN000034954) with the purpose to test the safety and tolerability of ropinirole hydrochloride in ALS patients [39].

Similarly, the anti-epileptic drug retigabine (ezogabine) (Figure 3) has been investigated in iPSC-derived MNs from ALS patients with mutations in *SOD1*, *C9ORF72,* and *FUS* [41,42,43]. As expected, ALS patient-derived iPSC MNs initially exhibited a hyper-excitable state, followed by a decrease in excitability when treated with ezogabine [41]. The results obtained in iPSCs elicited a double-blind, phase 2 randomized clinical trial with 65 enrolled patients that were treated with 600 mg/day or 900 mg/day of ezogabine or placebo for approximately 10 weeks (clinicaltrials.gov identifier: NCT02450552) [44].

Bosutinib is a Src/c-Abl inhibitor able to inhibit cell death, able to avoid the aggregation of misfolded SOD1 inducing autophagy. In a clinical trial using bosutinib in ALS subjects (UMIN000036295), the safety and tolerability of BOSULIF (bosutinib) tablets (100 mg/day, 200 mg/day, 300 mg/day, or 400 mg/day) were evaluated to determine the maximum tolerated dose and a recommended Phase II dose of bosutinib for treatment of ALS patients [39].

A previous transcriptomic study revealed the altered expression pattern of PACAP and VIP (two neuropeptides that were naturally expressed in brain) in motor cortex of a subgroup of ALS patients [45]. Given the well-known pleiotropic effects of PACAP and VIP in neuroprotection [46,47], several authors have suggested the use of PACAP in clinic to prevent evolving of neurodegenerative diseases [46,47,48,49,50,51]. Recently, by using an apoptotic iPSC-derived motor neuronal model, we have examined the trophic effects of exogenous PACAP following neurodegenerative stimuli, and demonstrated that treatment with 100 nm PACAP was able to effectively rescue iPSC-derived MNs from apoptosis, showing the efficiency of PACAP in enhancing the motor neuron viability and providing a proof-of-principle for ALS treatment [52] (Figure 3).

With advances in organoid technology, extensive 3D model systems can be generated to study ALS and optimize drug discovery and/or screening processes. For example, a recent study has reported the development of a 3D organoid model of ALS from both human iPSC-derived muscle fiber cells and human ESC-derived MNs from a patient with ALS harboring *TDP-43* mutation [53]. In another study, ALS organoids that were treated with rapamycin and bosutinib exhibited improved muscle contraction and motor neuron viability when compared to untreated organoids, supporting their use for candidate drugs screening and prediction of patient response to treatments [54].

## 4. iPSC-Based Drug Testing for Parkinson’s Disease

Parkinson’s disease (PD) is a common neurodegenerative disorder, with an increasing incidence in aged people [55]. It is mainly caused by the progressive loss of striatal-projecting midbrain dopaminergic neurons of the ventral forebrain, resulting in both motor (bradykinesia, rigidity, resting tremor, and postural instability) and cognitive (depression, dementia, hallucinosis, sleep, and sensory disorders) deficits. The disease has become a rapidly growing area of concern because of the high prevalence of PD and increase in the proportion of aging population resulting from extended life expectancy.

The majority of PD cases are sporadic with unknown etiology. However, an approximate 10% represent familial cases and are caused by monogenic PD forms. Mutations in the α-synuclein gene (*SNCA*), in the leucine-rich repeat kinase 2 gene (*LRRK2*), and Vacuolar protein sorting ortholog 35 (*VPS35*) account for the autosomal-dominant PD forms, while mutations in PTEN-induced putative kinase 1 (*PINK1*), Parkin (*PRKN*), and protein deglycase DJ-1 (*PARK7*) are responsible for the autosomal recessive forms [56]. Some genes are linked to the atypical recessive forms of PD (*ATP13A2*-PARK9, *PLA2G6*-PARK14, and *FBXO7*-PARK15) or X-linked (*ATP6A2* and *TAF1*), whereas others (*PARK3, UCHL1, PARK10, GIGYF2, PARK12, HTRA2, PARK16, EIF4G1, DNAJ, HLA-DR, GAK-DGKQ, SYNJ1,* and *GBAP1*) may influence PD susceptibility [57]. Despite the efforts in the field of PD therapies, there is still no therapeutic strategy capable of preventing or slowing down PD progression and reverting neurological disabilities. Only symptomatic or palliative treatments are available, such as dopamine-replacement drugs (Levodopa) or deep brain stimulation procedures.

**Figure 4:**
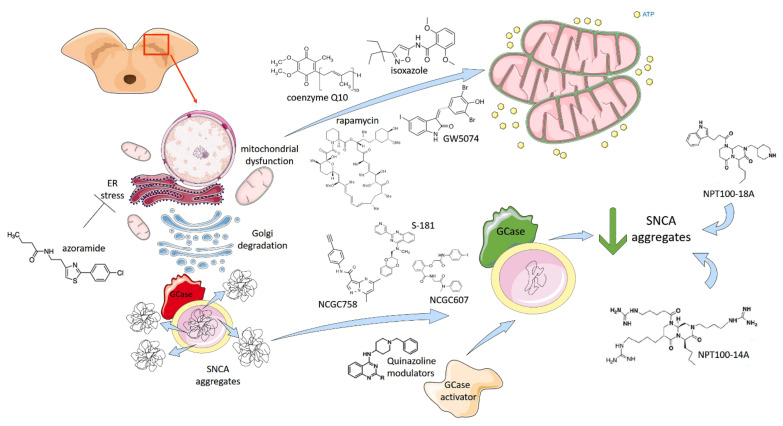
iPSC-based drug testing to treat Parkinson’s Disease. The main pathological characteristics of PD are the presence of Lewy bodies (aggregation and accumulation of the protein α-synuclein), which cause mitochondrial disfunctions, endoplasmatic reticulum (ER) stress and Golgi degradation, leading to cell death in the brain’s basal ganglia. Human iPSC-derived dopaminergic models have facilitated the study of PD, providing amenable systems for drug discovery as well. Promising results derive from drugs targeting the glucocerebrosidase (GCase) pathway. Inactive GCase (in red) can be restored in active GCase (in green) directly using GCase chaperones NCGC758, NCGC607 and novel small molecules, such as S-181, or indirectly using Quinazoline modulators able to trig GCase activator. These compounds reduce lysosome accumulation of α-synuclein in both wildtype and mutant GCase patient-specific iPSC-derived dopaminergic neurons. SNCA aggregates can also be directly targeted by small molecules NPT100-18A, NPT100-14A and their derivatives, which are able to revert the degenerative phenotype. Different drugs, such as isoxazole, coenzyme Q10, rapamycin and GW5074 (a LRRK2 kinase inhibitor) have been tested in iPSC-DA models showing effects in increasing mitochondria respiration and biogenesis. The stress of ER is another drug target to treat PD. A novel unfolded protein response (UPR) modulator, azoramide restore ER function in PLA2G6 D331Y mutant DA neurons. Illustrations used elements from Servier Medical Art (www.servier.fr/servier-medical-art).

Recent advances in cell reprogramming technologies have facilitated the generation of human iPSC-derived dopaminergic (DA) models for studying both familial and sporadic PD, supporting the identification of early pathological phenotypes and providing amenable systems for drug discovery (Figure 4).

Promising results arise from drugs targeting the glucocerebrosidase (GCase) pathway, as described by multiple studies. Two GBA chaperones, NCGC758 and NCGC607 (Figure 4), were found to restore GCase activity and reduce lysosomal substrate accumulation in multiple PD models of iPSC-DA neurons [58,59], while the conversion of quinazoline modulators by N-methylation resulted in the generation of GCase activators, partially stabilizing GCase activity and improving its activity in PD patient-derived fibroblasts and DA midbrain neurons [60]. The inhibition of acid ceramidase using carmofur decreased glucosylsphingosine levels in GCase-deficient cells and reduced α-synuclein accumulation in PD patient-derived DA neurons [61]. Finally, a recent work described a novel small-molecule modulator of GCase (S-181) able to increase wild-type GCase activity in iPSC-derived DA neurons from patients with 84GG-*GBA*, as well as in *LRRK2*, *Parkin*, *DJ1*-linked, or sporadic PD patients [62]. Thus, GCase activity represents a major target for PD therapeutic treatment that is associated with multiple forms of PD, including both genetic and idiopathic cases.

Further drugs have been tested in iPSC-DA models showing effects in ameliorating phenotypes. For example, the long-term treatment of iPSC-DA neurons from idiopathic and familial PD patients with the mitochondrial-targeted antioxidant mito-TEMPO or NAC blunted the accumulation of oxidized dopamine and improved lysosomal GCase activity and proteolysis [63]. The activation of the transcription factor *MEF2C* by isoxasole rescued α-synuclein p.A53T iPSC-DA neurons from nitrosative stress via the MEF2C-PGC1α pathway by increasing the mitochondria respiration and biogenesis [64]. Moreover, small molecules targeting α-synuclein oligomerization (NPT100-18A, NPT100-14A, and ELN48228) were described to revert the degenerative phenotype under both basal and induced stress conditions in iPSC-DA neurons harbouring the p.A53T mutation (Figure 4), indicating a treatment strategy for PD and other synucleinopathies [65]. Other drugs, such as coenzyme Q10, rapamycin, and the LRRK2 kinase inhibitor GW5074, were able to rescue cellular vulnerability that was associated with mitochondrial dysfunction in iPSC-derived neural cells from familial PD patients and at-risk individuals [66]. Recently, a novel unfolded protein response (UPR) modulator, azoramide, has been shown to exert protective action on mutant patient-derived midbrain DA neurons harbouring the homozygous phospholipase A2 group 6 (*PLA2G6*) D331Y mutation. In particular, azoramide treatment significantly protected *PLA2G6* D331Y mutant DA neurons against ER stress, abnormal calcium homeostasis, mitochondrial dysfunction, increased reactive oxygen species, and apoptosis via restoring the ER function and CREB signalling (Figure 4), which suggests this drug as a potential neuroprotectant against DA neurons damage [67].

Interesting opportunities derive from the cultivation of PD patient-specific neurons in 3D in vitro settings, where the cells are subjected to mechano-structural cues that bring them closer to physiological microfluidic conditions [68]. Starting work in this area found that 3D cultures of neurons carrying the *LRRK2*-G2019S mutation show mitochondrial abnormalities and cell death, an effect that is partially reverted by the administration of the LRRK2 inhibitor 2 (Inh2) [69]. Therefore, the possibility of using 3D in vitro testing to stratify PD patients for proper drug administration is a key opportunity in bringing the research work closer to personalized medicine for future clinical applications.

## 5. iPSC-Based Drug Testing for Fragile X Syndrome

Fragile X syndrome (FRAX) is an inherited genetic condition leading to cognitive disability and it is frequently associated to autism spectrum disorder (ASD) [70]. The incidence of FRAX in males is approximately 1:4000–7000 and in females is 1:4000–6000 worldwide [71]. FRAX is a monogenic disorder that is related to a loss-of-function mutation. In particular, the fragile X mental retardation 1 (*FMR1*) gene contains an expansion of the cytosine-guanine-guanine (CGG) triplet repeat located within the 5′ untranslated region (UTR) of the gene [72]. Deletions and sequence variants within FMR1 result in a very small fraction of FRAX cases. The number of CGG repeats is highly polymorphic, healthy individuals have between 6–54 repeats, with 29 or 30 repeats being the most common allele. Alleles with 45–54 CGG repeats are referred as intermediate alleles; if the number of repeats expands between 55–199, they are considered to be premutation (PM) alleles that are usually unstable and easily expand to full mutations alleles (FM). Finally, alleles with more than 200 repeats (FM) present a hypermethylated FMR1 promoter, which prevents the expression of FMR1 [73]. FMRP is a highly conserved RNA-binding protein, which is ubiquitously expressed in mammals, especially in the brain and testes. It plays a key role in the transport, stabilization, and translation of messenger RNA (mRNA) into proteins that affect neuronal development, function, and synaptic plasticity [74]. To date, most efforts have been directed towards the development of drugs for FRAX that may reduce the signs and symptoms of the disease rather than dealing with its cause (FMRP deficiency). However, scientists are aiming to develop alternative therapeutic approaches to treat patients by developing strategies to re-activate the FMR1 gene and restore its function. To this end, iPSCs provide a powerful platform for the study of the molecular mechanisms that are involved in FRAX and drug development [75,76].

**Figure 5:**
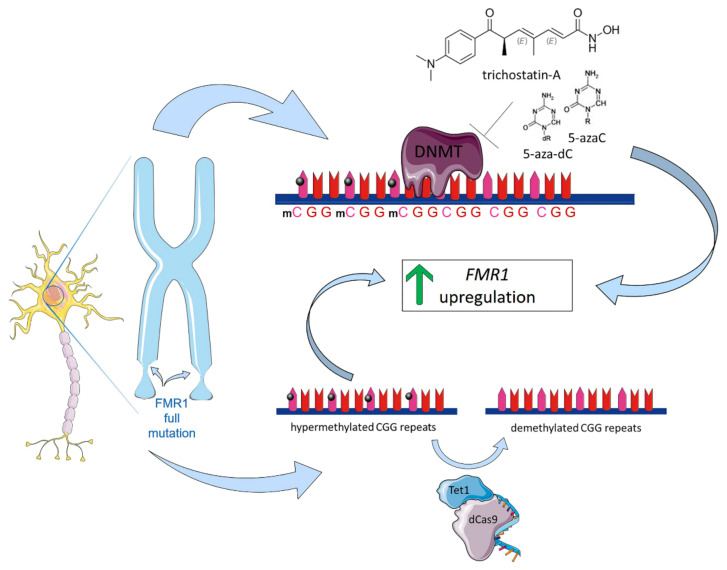
iPSC-based drug testing to treat Fragile X Syndrome. FRAX is a monogenic disorder related to a loss-of-function mutation type of *FMR1* gene containing an expansion of the cytosine-guanine-guanine (CGG) triplets repeat located within the 5′ untranslated region (UTR) of the gene. Alleles with more than 200 repeats, considered full mutated alleles (FM), present the FMR1 promoter hypermethylated, which prevents the expression of FMR1 due to epigenetic gene silencing. iPSCs provide a valuable instrument for developing gene therapy-based approaches to FRAX. Different chemical compounds have been studied to remove epigenetic marks. To date, two approaches have been tested. The first one investigated the effect of chromatin remodeling compounds, such as trichostatin-A (TSA), 5-azacytidine (5-azaC), and 5-aza-2′-deoxycytidine (5-aza-dC). These compounds lead to the loss of DNA methylation by the inhibition of DNA methyltransferases (DNMT), restoring FMR1 expression in FRAXA iPSCs and their neural derivatives. The second approach was based on dCas9-Tet1/single guide RNA tool, a new developed DNA methylation editing technology that induces demethylation of the CGG expansion, switches the heterochromatin status of the upstream FMR1 promoter to an active chromatin state, and restores a persistent expression of FMR1 in FRAXA iPSCs. Illustrations used elements from Servier Medical Art (www.servier.fr/servier-medical-art).

FRAX iPSCs-derived neurons are particularly informative since they retain epigenetic memory and, therefore, are always completely methylated, manifesting the final state of gene inactivation in the patients’ somatic cells. In addition, iPSCs provide a valuable instrument for developing gene therapy-based approaches to FRAX. Over the last few years, several studies have used iPSCs-derived neurons to evaluate chemical compounds that are capable of removing the epigenetic marks. Bar-Nur et al. investigated the effect of chromatin remodeling compounds, including the general histone deacetylase inhibitor trichostatin-A (TSA) and the de-methylating agents 5-azacytidine (5-azaC) and 5-aza-2′-deoxycytidine (5-aza-dC), which, at low doses, lead to the loss of DNA methylation by the inhibition of DNA methyltransferases (DNMTs) and restoring FMR1 expression in FRAX-iPSCs and their neural derivatives (Figure 5) [77].

Dan Vershkov et al. [78] showed the induction of FMR1 mRNA expression in FRAX-iPSCs following treatment with DNMT inhibitors. More recently, Shawn Liu et al. applied a new developed DNA methylation editing technology (dCas9-Tet1/single guide RNA) to reverse the hypermethylation of CGG repeats in the FMR1 gene (Figure 5). Their results showed that the targeted demethylation of the CGG expansion switched the heterochromatin status of the upstream FMR1 promoter to an active chromatin state, which restored a persistent expression of FMR1 in FRAX iPSCs.

## 6. Conclusions: Challenges and Perspectives for iPSC Use in Drug Screening

Somatic cell reprogramming into iPSCs has brought disease modeling towards new standards by capturing the single patient’s genome, and it currently represents a powerful system for high-throughput drug screening and personalized drug discovery [79,80]. Properly developed iPSCs-based models own the potentiality to faithfully mimic human molecular and cellular conditions, and they are a valid complementary method to in vivo animal models. iPSC-derived neurons, in particular, offer the possibility of drug screenings in neuronal cells with the exact genetic profile of patients with a particular disease. This provides an opportunity to test target compounds for patients with different genetic backgrounds, which enables the identification of molecules that could be beneficial for patient subgroups, facilitating precision medicine for neurological diseases. However, limitations regarding both the establishment of robust differentiation methods and the large variability obtained in cell cultures still need to be addressed [81]. The culturing and maintenance of iPSCs, as well as their differentiation towards neuronal populations, usually require long-term and expensive practices. It is difficult to keep cells in a pluripotent state, and small changes in the culturing conditions can lead to unwanted spontaneous differentiation or to a reduction in proliferation. Moreover, these protocols often require expensive recombinant proteins, such as cytokines or specific growth factors, and they need additional interventions to induce the effects of aging and the exposure to toxic stressors. A further relevant technical limitation relies on the large heterogeneity of iPSCs cell lines obtained from different batches and laboratories. The development of better growth substrates and media, together with the use of automated systems could improve the reliability of iPSC culture, reducing spontaneous differentiation and enhancing survival [82]. In addition, some studies have introduced 3D organoid-based technologies that better mirror an organ’s biological architecture, endogenous signaling, and intercellular interactions with respect to 2D model cultures. Although this technology is still in its infancy, the use of stem cells in drug discovery opens a wide spectrum of possibilities in the field of neurological disorders, offering both a cost-effective approach and paving the way for personalized medicine.

In conclusion, the development of new drugs is a complex and expensive procedure. To date, the screening of pharmacological compounds libraries and repurpusing of drugs strongly rely on preclinical models. For complex and multifactorial neurological diseases, such as those described above, the rarity of biological material does not easily allow the development of suitable human-based models. To this end, the ability of iPSC derived cell models to differentiate towards specific neural lineages opens a new possibility to obtain a unique and unlimited platform that recapitulates in vitro single patients’ aspects of human neurological diseases for personalized drug testing.

Future efforts must be directed towards the optimization of reprogramming conditions, such as the efficiency of lineage-specific reprogramming, the ability to obtain stable systems for large scale screenings, and the implementation of automated and large-scale systems in order to take advantage of the full spectrum of possibilities that are offered by iPSCs.

## Data Availability

Not applicable.
